# Strengthening adaptive functioning and participation in the contemporary spinal muscular atrophy paradigm: clinical perspectives and future directions

**DOI:** 10.1007/s12519-026-01063-0

**Published:** 2026-07-22

**Authors:** Emily E. Farah, Michelle A. Farrar, Didu S. Kariyawasam, Sarah-Grace Paguinto

**Affiliations:** 1 Discipline of Pediatrics and Child Health, School of Clinical Medicine, UNSW Medicine and Health, University of New South Wales, Sydney, NSW Australia; 2 Department of Neurology, Sydney Children’s Hospital Network, Sydney, NSW Australia; 3 Neuroscience Research Australia, UNSW, Sydney, NSW Australia; 4 Occupational Therapy Department, Sydney Children’s Hospital, Randwick, NSW Australia

**Keywords:** Adaptive functioning, Children, International classification of functioning, Patient-reported outcome measures, Pediatrics, Spinal muscular atrophy

## Abstract

**Background:**

The developmental trajectory for children with spinal muscular atrophy (SMA) has been significantly altered following the availability of survival motor neuron (SMN) augmenting therapies. In the treatment era, a holistic patient-centred approach that looks beyond outcomes of survival and motor function is critical and must incorporate everyday functioning (“adaptive functioning”) and participation.

**Methods:**

A literature review was conducted using PubMed, drawing on articles published until 2025. Keywords included: “spinal muscular atrophy”, “adaptive function”, “adaptive behaviour”, “the International Classification of Functioning, Disability and Health”, “function”, “participation” and “patient-reported outcome measures”. Articles were screened and included on the basis of their contextual relevance to the current SMA care paradigm, with preference given to studies providing insights into the application of the International Classification of Functioning, Disability and Health (ICF), and adaptive functioning.

**Results:**

This review highlights the growing importance of everyday functioning and participation within the contemporary SMA treatment paradigm. Current evidence suggests that adaptive functioning is affected across mobility, self-care, social, and neurodevelopmental domains. The ICF provides a practical and multidimensional framework that supports a holistic assessment approach, extending beyond traditional performance-based and motor-focused outcomes to better capture capabilities in everyday functioning, including outcomes that directly influence independence, participation, and quality of life for children with SMA and their families. Furthermore, the ICF may assist in identifying treatment goals, guiding personalised management, and facilitating multidisciplinary collaboration. A limitation of current evidence is that thus far studies have predominately been completed prior to the advent of SMN augmenting therapies, emphasising the necessity of assessing adaptive functioning, especially in the domain of patient-reported outcome measures, to support personalised care and meaningful long-term outcome assessment.

**Conclusions:**

The ICF is highly applicable for applying a holistic management approach for SMA in the treatment era, enabling SMA to be conceptualised through a multidimensional lens. Developing a minimum set of core SMA outcomes which incorporate the ICF framework and aim to understand and improve outcomes is a priority and of practical relevance to people with SMA, families and clinicians. This approach promotes an essential paradigm shift by emphasising functioning and participation as key focuses within clinical care and research.

**Graphical Abstract:**

**Figure:**
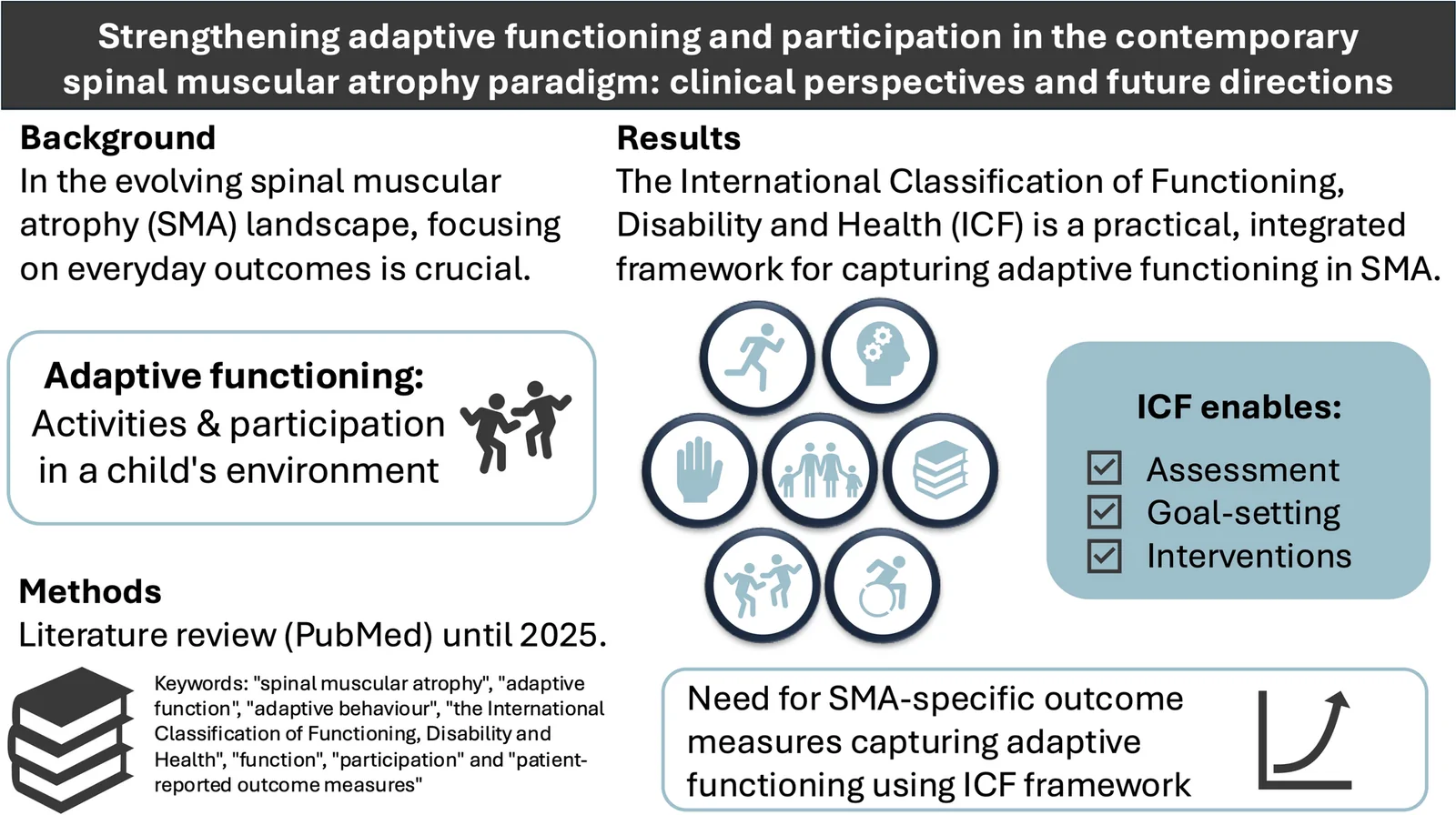

## Introduction

Spinal muscular atrophy (SMA) is a rare neuromuscular disorder affecting 1 in 10,000 individuals [1]. It is caused by biallelic pathogenic variants in the survival motor neuron 1 gene (*SMN1*), leading to a deficiency of the ubiquitously expressed survival motor neuron (SMN) protein [2]. Age of onset and severity are inversely associated with copy numbers of the paralogous survival motor neuron 2 gene (*SMN2*) [2]. The pathogenesis leads to irreversible degeneration of alpha motor neurones from the spinal cord and brainstem, leading to muscle atrophy and weakness, accumulation of neurodisability and comorbidities, and loss of function.

The SMA has historically been classified into different types, based on the age of onset and the maximum motor milestones attained. This ranges from foetal (type 0, most severe), to onset before 7 months (type I, non-sitter), between 7 and 18 months (type II, sitter), after 18 months (type III, walker) and during adulthood (type IV, mildest) [3–5]. The early diagnosis and treatment of SMA with SMN augmenting therapies have transformed health outcomes and a classification based on current motor function (non-sitters, sitters, standers, and walkers) is now utilised to guide best practice, with the measurement of motor function being a central focus in clinical trials to demonstrate the magnitude of treatment effects (Table 1) [6, 7].

**Table 1:** Motor function scales utilised in phase III global clinical studies

Motor function scale	Comments
Non-sitters	
CHOP–INTEND	There are 16 items assessing movement, hand grip, limb flexion or extension and head stabilisation. Created for SMA type I and non-sitting infants with neuromuscular conditions
HINE-2	Evaluates motor milestones and incremental changes for people with no or minimal achievements in attaining milestones. Can compare milestones between people with SMA and normative cohorts
Sitters	
HFMSE	Reports gross motor function in 33 items. Aiming to incorporate function in people with SMA type II and III
RULM	Assesses upper limb function using a range of activities reflective of daily tasks, including elevating a cup and drawing on paper. Can rate distal muscle strength
MFM-32	Assesses motor functional abilities in three domains across 32 items: standing and transfers, axial and proximal motor function, and distal motor function
Ambulant	
6-minute walk test	Enables assessment of gait, endurance, and fatigue in walkers
HFMSE	
RULM	
All	
WHO motor milestones	Assesses the six major gross motor milestones with a window period for achievement

*SMA* spinal muscular atrophy, *CHOP–INTEND* Children’s Hospital of Philadelphia infant test of neuromuscular disorders, *HINE-2* Hammersmith infant neurological examination—part 2, *HFMSE* Hammersmith functional motor scale—expanded, *RULM* revised upper limb module, *MFM-32* motor function measure, *WHO* World Health Organization

Scales utilized in Phase III global clinical studies and suggested by the most recent standards of care [6, 7]

While motor function assessments are important, people with SMA emphasise the imperative for functional outcomes with direct everyday relevance, necessitating a holistic assessment approach that can capture the full spectrum of phenotypes and the multidimensional impact of SMA within the new treatment landscape [8–10]. Efforts have thus far focused upon reporting health-related quality of life (HRQoL), encompassing the physical, psychological, and social-cognitive factors that affect well-being. A critical yet underexplored facilitator of HRQoL is adaptive functioning, which is an individual’s capability to navigate their personal needs in everyday life and actively participate in their environment [11]. This corresponds with standards of independence expected for the individual’s age and environmental context, and incorporates neurodevelopmental factors, neurocognition and social-communication skills, which are key determinants of functioning. As global research continues to expand evidence for the outcomes achieved by children in the new SMA paradigm, adaptive functioning represents an important marker of treatment efficacy.

**Fig. 1:**
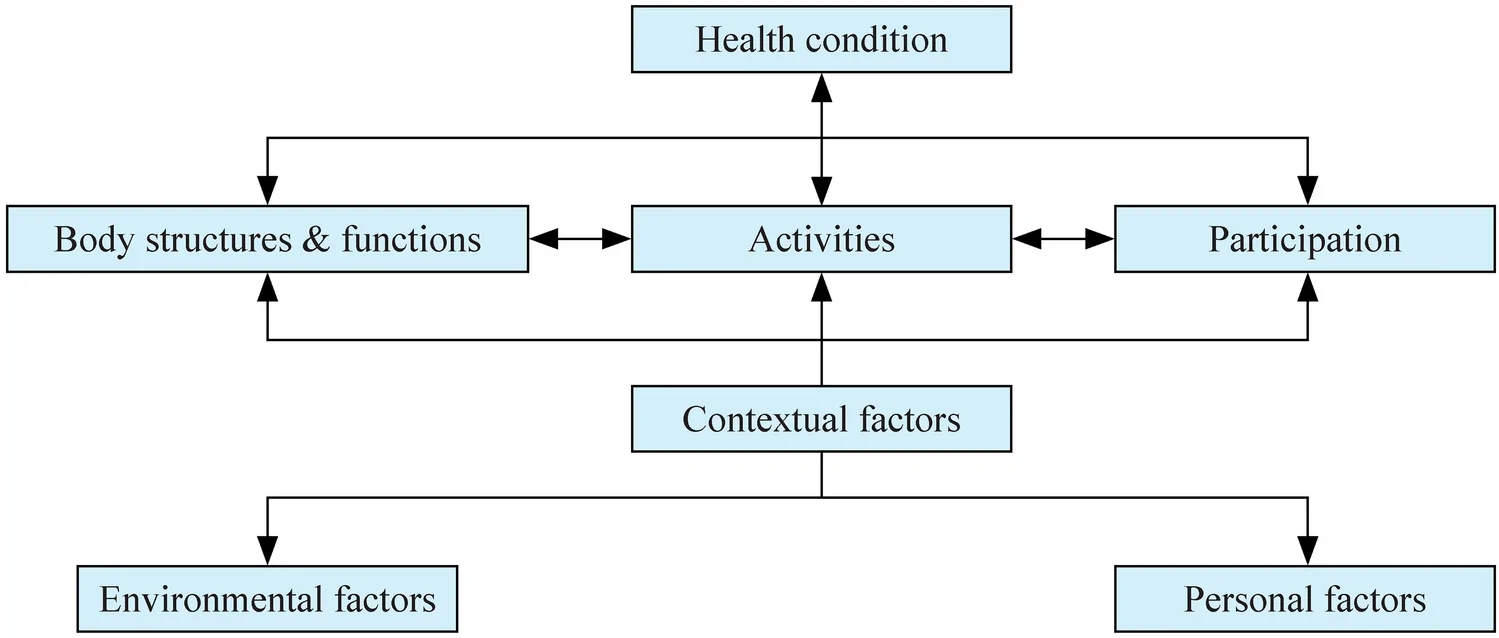
International Classification of Functioning, Disability and Health (ICF) framework. This is an adaptation of an original work “ICF: International classification of functioning, disability and health. Geneva: World Health Organization (WHO); 2001. Licence: CC BY–NC–SA 4.0”. This adaptation was not created by the World Health Organization (WHO). The WHO is not responsible for the content or accuracy of this adaptation. The original edition shall be the binding and authentic edition

The World Health Organization’s (WHO) International Classification of Functioning, Disability and Health (ICF) provides a framework for the assessment of adaptive functioning in SMA. It describes an individual’s everyday functioning as the interplay of health and contextual factors [12]. The ICF is composed of three core components, body functions and structures, activity, and participation, to holistically depict health as a dynamic, interconnected web (Fig. 1) [13]. The ICF represents a merger of the medical and social models of health; the medical model being traditional perspectives that disability results in impaired functioning, whereas the social model perceives society to place limits on an individual’s functioning. This merger is achieved through activity, which is required by both models to determine the impacts of disability or the environment on functioning [14]. Adaptive functioning is central to the ICF, encapsulating the “activity” and “participation” domains [12]. This description of functioning is aided by the ICF’s distinction between capacity and performance. Capacity refers to an individual’s best functioning captured within clinical environments using standardised tools, whereas performance is an individual’s typical functioning within their daily environment which is captured through self-reported assessment. Through comparing an individual’s reported capacity and performance, the influence of the environment upon functioning can be assessed and optimised [15, 16]. Using the ICF coding system, the ICF can be applied as a direct classification system or used to generate novel assessment tools and goals for children with rare, complex and chronic conditions, and thus has important potential for capturing outcomes, formulating priorities and personalised interventions in the new SMA era. The aim of this review was to synthesise existing literature regarding the ICF as a multidimensional, integrated framework for clinicians to assess and optimise everyday participation and functioning in children with SMA. Furthermore, this review aimed to highlight its relevance and application in the contemporary era of SMA therapies, to facilitate the provision of person and family-centred care.

### Methods

A targeted literature search strategy was completed on PubMed using a combination of keywords and Medical Subject Headings (MeSH) terms. The search strategy keywords included: “spinal muscular atrophy” combined with “adaptive function”, “adaptive behaviour”, “the International Classification of Functioning, Disability and Health”, “function”, “participation” and “patient-reported outcome measures”. Full-text articles published in English up to July 2025 were considered for inclusion. Manual searches of reference lists were also conducted to include additional full-text articles. Articles were included on the basis of their contextual relevance to the current SMA care paradigm, with articles regarding survival and prognosis in the era prior to SMN augmenting therapies excluded. Preference was given to original research articles which were of direct clinical relevance for optimising care (relevant to the patient, actionable and pertaining to clinical outcomes). Studies providing insights into the application of the ICF, adaptive function and participation, meaningful outcomes, and the use of patient-reported outcome (PRO) measures for people with SMA were prioritised.

## Results

### International classification of functioning, disability and health and its application to children with spinal muscular atrophy

The ICF has been applied to cohorts of children with diverse physical disabilities, including people living with SMA who have not received SMN augmenting therapies, and found to be a valid, reliable framework to assess functioning and necessary environmental supports [17–20]. It has also been reported to facilitate the individualisation of supports that are not revealed through a medical model of assessment in children receiving SMN augmenting therapies. One proof-of-concept study for a child (19 months) with SMA type II utilised a set of ICF codes as a scoring tool at treatment initiation and 6 month post-initiation to identify the child’s functional goals, guide multidisciplinary management and monitor improvements [21]. At these timepoints, scores for the ICF codes were informed by clinical evaluation and standardised assessments, including the Hammersmith Infant Neurological Examination-Part 2 (HINE-2), Hammersmith Functional Motor Scale (HFMSE), Functional Independence Measure for Children (WeeFim) and Pediatric Quality of Life Inventory (PedQL) [21]. This study found improvements in activity and participation scores following access to environmental interventions (i.e., SMN augmenting treatment and rehabilitation) and determined the ICF could be used to guide holistic management in the era of SMN augmenting therapies.

These findings were supported by a second study which captured patterns of functioning in six adolescents with SMA type II and III using a standardised interview assessing a set of ICF codes [22]. The study reported impairments in bodily functions, including motor, respiratory and bulbar functions, and challenges in daily activities and participation within the cohort, notably in completing activities of daily living, such as self-care, eating and dressing. In addition, environmental influences identified by participants, such as accessible transportation, equipment access, and government support, were demonstrated to either improve or exacerbate functional limitations. Together, the studies emphasise the multidimensional nature of SMA and the usefulness of the ICF in analysing complex factors to develop personalised multidisciplinary care approaches.

### Adaptive functioning in children with spinal muscular atrophy

Two primary studies have evaluated adaptive function in children with SMA in the era before SMN augmenting therapies, both identifying limitations in functioning and supporting its relevance as an outcome measure [23, 24]. One SMA natural history study incorporated assessment of functioning in a subgroup of 39 children (four with SMA type I) using the WeeFim, assessing self-care, cognition and mobility [23]. Over 90% required mobility assistance, with stair management a consistent barrier to independence. All children with SMA type I and the majority with type II (73%) reported needs for self-care assistance, especially bathing and dressing. However, WeeFim has not been validated for SMA, and as a self-report measure is susceptible to socially desirable responding. Furthermore, the low response rate and attrition bias of the cohort meant such results could have underestimated functional difficulties. Challenges in self-care and mobility were corroborated in a study of adaptive functioning and mental health in 50 children with neuromuscular diseases, including 14 children with SMA (2.4–13.6 years) [24]. The communication, cognitive, social and self-direction skills of the SMA cohort matched normative reference data, although health and safety, home living and self-care skills were impaired. Together, these historical studies support the measurement of adaptive functioning in care models and provide insights into adaptive functioning in small SMA populations, although the external validity of these studies in the era of SMN augmenting therapies is limited.

In the era of SMN augmenting therapies, few published studies evaluate adaptive functioning in children with SMA receiving SMN augmenting therapies. A study by Lee et al. [25] incorporated adaptive functioning in a longitudinal assessment of the impact of nusinersen treatment on HRQoL and caregiver burden among people with SMA type II and III (*n* = 24, 6.8–269.4 months) using the pediatric evaluation of disability inventory computer adaptive test (PEDI–CAT). This tool detected significantly meaningful improvements in responsibility and social-cognitive domains, but no significant changes in mobility or daily activity over time with nusinersen. Studies of the neurocognitive profile of children with SMA type I (*n* = 15, 0.0–6.1 years) have incorporated adaptive functioning using the Vineland Adaptive Behaviour System—Second Edition and Griffiths Scales of Child Development 3rd Edition (Griffiths III) and psychological outcomes including anxiety, mood, coping strategies and parental stress [11]. Global difficulties in adaptive functioning were recorded, with an adaptive behaviour composite of 68.67, and specific weaknesses in motor skills (58.40) and daily living skills (68.73). Furthermore, the VABS-II general adaptive behaviour composite score aligned with results from the Griffiths III general development quotient, demonstrating significant global adaptive deficits. These studies are consistent in their description of low adaptive functioning in treated SMA type I cohorts, underpinned predominantly by weaknesses in motor function and daily activities. Although these scales were useful in mapping functional strengths and challenges, the VABS-II and Griffiths III have not been validated for use in SMA.

Furthermore, a recent study explored the functional independence and mental health of school-aged children with SMA type II and type III (8.73–11.90 years) receiving nusinersen, through longitudinal assessment from treatment initiation to 14 months [26]. Utilising a battery of assessments, including the SMA independence scale-upper limb module (SMAIS-ULM), the study determined that significant improvements in independence resulted following treatment initiation, alongside improvements in anxiety and depression observed amongst the cohort.

#### Neurodevelopment in the era of survival motor neuron augmenting therapies

Early neurodevelopment is an important prelude to neurocognitive function in childhood and throughout the lifespan, influenced by the interplay of biological factors, early experiences, and environment [27]. Neurocognitive function, encompassing important skills such as everyday cognition, behavioural regulation and language-based outcomes, is critical for understanding and engaging with the daily environment and adapting to meet daily needs, although it is commonly reported independently of adaptive functioning [11]. The potential for long-term neurocognitive sequelae is an important consideration for the emerging cohort of phenotypically diverse children with SMA type I who have received SMN augmenting therapies. Here, variations in the neurodevelopmental profile of children with SMA are emerging, who, prior to the emergence of disease-modifying therapies and implementation of newborn screening, would not survive to the age of developing language skills (or speak due to tracheostomy placement) [5]. Clinical studies have identified neurodevelopmental differences in small single-centre cohorts of children treated with SMN augmenting therapies, including cognitive, expressive language and communication performance [28–31]. Studies utilising general pediatric neurodevelopmental tools such as the Griffiths III and Bayley Scales of Infant and Toddler Development third edition (BSID-III) have shown deficits in language abilities, alongside autism spectrum disorder [11, 32–34]. Furthermore, while neurodevelopmental comorbidities are cited in case reports and to varying degrees in the existing literature, one international survey determined that of the 43% of participants reporting a neurodevelopmental comorbidity, only 30% had undergone formalised assessment for neurodevelopmental disorders [35]. Current findings concerning neurodevelopment are inconclusive and such wide variability likely reflects methodological challenges, namely, a lack of age appropriate, SMA-specific measures which can isolate and separate motor-related weakness from neurodevelopmental disorders. Furthermore, longitudinal assessments are important to identify the complex interplay between cognition and motor function in shaping receptive and expressive language development, including vocabulary, oral motor skills, articulation and vocal projection.

### Capturing adaptive functioning in children with spinal muscular atrophy: the future

There is growing recognition of the need to develop statistically robust SMA-specific PRO measures which capture adaptive functioning and facilitate clinical assessment in alignment with the ICF [36].

#### Current patient-reported outcome measures for adaptive functioning in spinal muscular atrophy

There are few current disease-specific tools which assess adaptive functioning in children with SMA, and even fewer are validated to sensitively detect longitudinal changes in adaptive functioning or have utility with children under 12 years (Table 2) [37]. To date, PEDI-CAT is the only norm-referenced PRO measure validated for use in pediatric SMA cohorts which specifically assesses adaptive functioning. PEDI-CAT is a generic caregiver-reported tool to assess adaptive functioning for a range of abilities in pediatric populations and strongly represents the activity and participation domains of the ICF [39]. PEDI-CAT has high user acceptability, with 92.1% of respondents in one study of 96 participants agreeing that the mobility and daily activity domains provided meaningful information [40]. A Rasch analysis of PEDI-CAT in an SMA cohort prior to the advent of SMN augmenting therapies demonstrated PEDI-CAT was informative in assessing mobility in individuals with SMA type II and III, and additionally daily activities in SMA type III [41]. Floor effects occurred among children with SMA type I in the mobility and daily activities domains, as PEDI-CAT was unable to sensitively discriminate changes for children who could not independently sit or stand [41]. However, with the advent of SMN augmenting therapies and acquisition of new motor skills in children with SMA, it is anticipated these floor effects may not be observed in those with early diagnosis.

**Table 2:** Patient-reported assessments of adaptive functioning used in SMA

Tool	Age (y)	SMA type	Comments	Validation for SMA
VABS-II	0–90	All	Generic tool for measuring individual adaptive behaviour across communication, daily living skills, socialisation, and motor skills	No [38]
PEDI-CAT	0–20	All	Generic tool assessing daily activities, mobility, social/cognitive and responsibility. Useful for mobility and daily skills in SMA type II and III	Yes [39–41]
Griffiths III	0–5^*^	All	Generic developmental tool measuring foundations of learning, language and communication, eye and hand co-ordination, personal–social–emotional, and gross motor domains of learning	No [42]
PedsQL NMM Version 3.0	> 2	All	Assesses HRQoL, used as a proxy for adaptive functioning. Age-adjustable for developmental appropriateness	Yes [43, 44]
WeeFIM	> 3	All	Generic tool for assessing six functional independence domains	No [45]
ACTIVLIM	6–80	All	Determines activity limitations in neuromuscular diseases	No [46, 47]
SMAIS-ULM	> 2	II, III	Assesses independent functional skills and activities of daily living for SMA type II and non-ambulant type III. Caregiver-reported for individuals aged 2 y, and patient-reported for individuals aged over 12 y	Yes [48]
SMA-HI	> 12	II, III	A total of 15 domains of functioning, mainly focused on HRQoL	Being validated
EK2	> 12	II, III	Designed for non-ambulant DMD patients. Questionnaire for wheelchair user functional ability	No [49]
Barthel Index	> 12	II, III	Generic assessment of 10 activities of daily living, designed to assess baseline functioning	No [50]

*SMA* spinal muscular atrophy, *VABS-II* Vineland adaptive behaviour scales, second edition, *PEDI-CAT* pediatric evaluation of disability inventory computer adaptive test, *PedsQL NMM Version 3.0* pediatric quality of life inventory neuromuscular module, *HRQoL* health-related quality of life, *WeeFIM* the functional independence measure, *ACTIVLIM* activity limitations questionnaire, *SMAIS-ULM* SMA independence scale–upper limb module, *SMA-HI* SMA health index, *EK2* the Egen Klassifikation 2 scale, *DMD* Duchenne muscular dystrophy

^*^Griffiths until 5 y, 11 mon of age

Scales suggested by Messina et al. [51] and Muni-Lofra et al. [37]

The SMAIS-ULM is a PRO tool which assesses independent living skills and activities of daily living for children and people with SMA type II and III [52]. The SMAIS-ULM has been used in a clinical trial assessing the efficacy of risdiplam in people with SMA type II and type III (non-ambulant) [53]. Furthermore, clinical trials and single-site studies have utilised other (performance-based) general pediatric assessment tools to capture important elements of adaptive functioning including neurodevelopment, language and cognition [28, 30]. General neurodevelopmental assessment tools utilised in such studies include Griffiths III, BSID-III and VABS-II [54]. Notably, the motor and cognitive subscales of the BSID-III were included in the clinical outcome assessments for a clinical trial of presymptomatic infants receiving risdiplam [55]. The latter includes items, such as attention to familiar and unfamiliar objects, looking for a fallen object and pretend play and has reported abilities similar to age-matched children without SMA. However, such scales are not SMA-specific and cannot appropriately distinguish between true neurodevelopmental comorbidities and the sequelae of motor weakness, such as expression of language, limiting their usefulness in capturing an important element of adaptive functioning.

#### Co-designing meaningful patient-reported outcome measures for spinal muscular atrophy

**Fig. 2:**
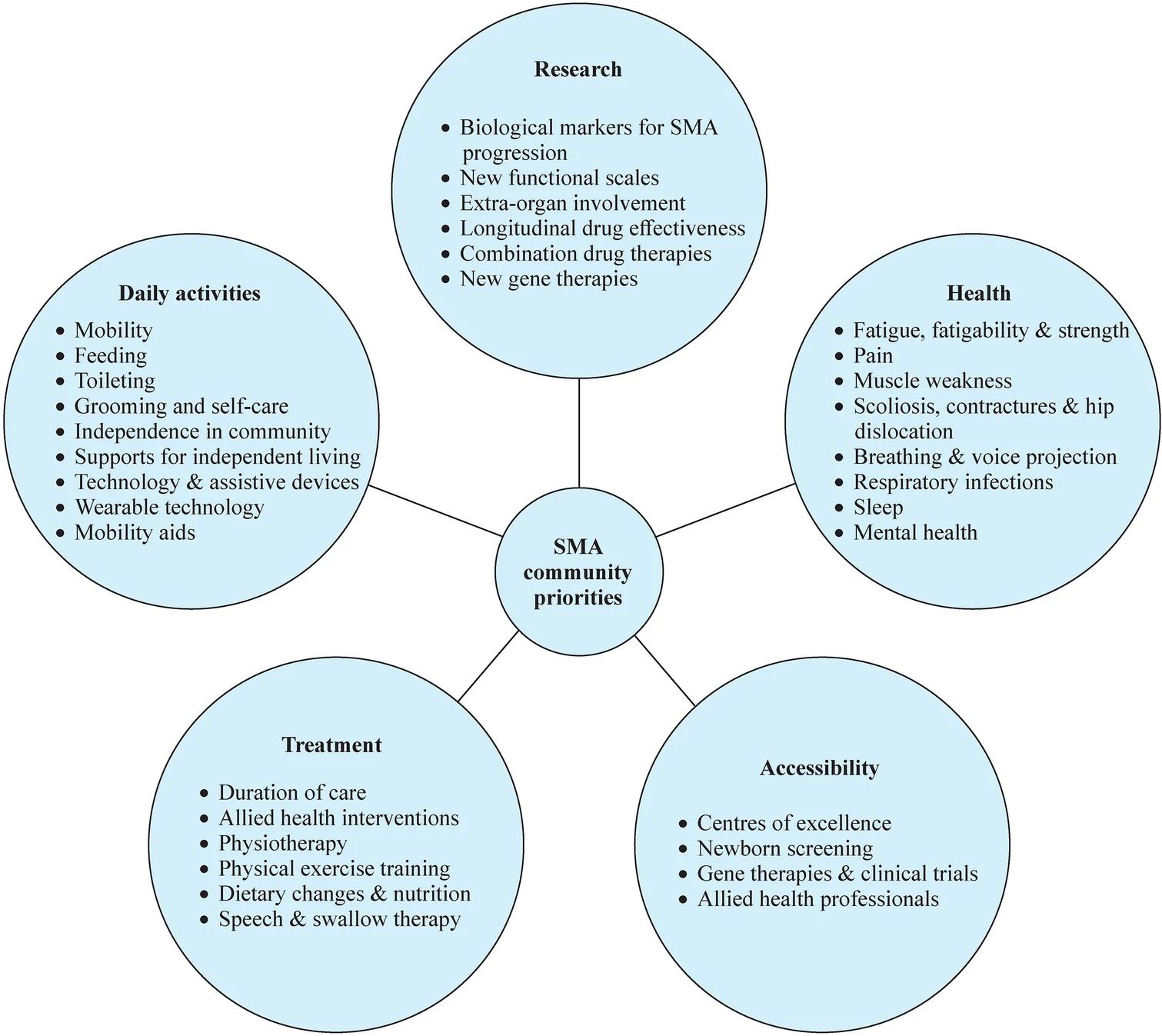
Research and clinical practice priorities for the spinal muscular atrophy (SMA) community. Community priorities are summarised from the PROfuture project, James Lind SMA Priority Setting Partnership study and Patient Reported Impact of Symptoms in SMA (PRISM-SMA) study. *SMA* spinal muscular atrophy

There have been recent efforts to identify SMA community priorities to inform the creation of meaningful PRO measures, which prioritise relevant outcomes (Fig. 2). These have identified functioning as an outcome of heightened importance. The PROfuture project represents an important step in developing PRO measures which capture adaptive functioning in SMA, aiming to determine the major domains affecting people with SMA [9]. Using five semi-structured focus groups with caregivers and people with SMA aged ten and above, the study identified ten core areas to incorporate in PROs assessing SMA burden: the primary areas being fatigue and fatiguability, pain, scoliosis and contractures, feeding, and breathing. Participants in the PROfuture project also emphasised that current performance-based scales cannot capture the impact of SMN augmenting treatments upon everyday functioning. These results were supported by a study which found that functional ability was the major determinant of meaningful change for people with SMA and caregivers [56]. Participants agreed motor function scales used as a proxy for functioning lacked sensitivity to identify small but meaningful functional changes, and measuring outcomes such as everyday activities, fatigue and swallowing were important. These priorities remain particularly important given the emergence of evidence suggesting sustained effects of fatigue and impaired bulbar integrity amongst children who have received SMN augmenting therapies [57]. Furthermore, a recent workshop of clinicians, people with SMA and advocacy groups targeted PRO measures in SMA and agreed that research should shift from HRQoL to assessing meaningful changes in everyday functioning [58]. Participants emphasised that current PRO tools assessing function were constructed prior to the advent of SMN augmenting therapies, exhibiting ceiling effects, necessitating new assessment methods to guide personalised interventions to optimise functioning.

## Discussion

On the eve of the first decade of a therapeutic revolution for SMA care, research and clinical priorities are undergoing major transformation. In the era of SMN augmenting therapies, the historical priorities of progressive motor loss, respiratory and nutritional care are rapidly shifting to instead address survivorship and long-term determinants of quality of life, expressly, everyday function and participation. This is especially important as the first cohorts of children diagnosed through newborn screening begin participating in school and the community, necessitating a robust evidence base to inform suitable supports. As the developmental trajectory of children with SMA continues to evolve in real time, a major challenge remains in predicting, measuring and optimising adaptive functioning and participation at home, school and in the community. Capturing adaptive functioning through the lens of the ICF framework by using appropriate PRO and performance-based tools is an important step in orientating the future of SMA care and necessitates further investigation and incorporation in research and clinical environments.

Describing the adaptive functioning and participation of children with SMA is crucial to answer the important questions of clinicians, caregivers and people with SMA regarding the long-term trajectory of SMA care [58]. Real-world studies have begun to define the diversity of adaptive functioning and apply the ICF framework, yet current gaps exist in understanding specific areas of strength and weakness for children with SMA, alongside environmental modifiers of adaptive functioning. In addition, important determinants of adaptive functioning identified in the pre-therapeutic era, namely, physiological fatigue and caregiver stress, have not been sufficiently explored in a post treatment paradigm. For example, fatigue is a common, complex and poorly characterised SMA symptom directly impacting everyday participation, level of functioning and quality of life, often limiting motor function and participation for individuals due to known effects on gait and mobility [59]. Qualitative studies of individuals with SMA and their families have endorsed this concept and have been consistently linked to fears of losing motor function and independence, and limited participation in everyday activities [9, 10]. As a predominant SMA symptom, it is increasingly important to characterise the broad and substantial impacts of fatigue upon adaptive functioning. Furthermore, parent/caregiver stress was identified prior to the advent of SMN augmenting therapies as an important environmental factor affecting the capacity of parents to support their child in achieving functional gains, with moderate to high levels of stress stemming from the psychological, physical, social, and financial demands of caregiving for children with SMA [60, 61]. Studies which capture adaptive functioning, assess environmental influences and identify modifiable factors associated with long-term differences in adaptive functioning amongst children with SMA are crucial to advocate for personalised care.

Clinical integration of PRO measures in research and clinical environments is essential to enable assessment of adaptive functioning in cohorts of children with SMA who have received SMN augmenting therapies. This can fill a knowledge gap pertaining to the everyday functioning of children with SMA and guide child- and family-centred rehabilitation SMA care to optimise outcomes. The use of PRO measures is pertinent to assess individual perspectives of health and functioning, with SMA groups advocating for increased collaboration to address community priorities [8, 36]. Such measures fulfil the expectations of clinicians and consumers to gather meaningful data addressing the SMA community agenda, reflected in their increasing clinical use [62, 63].

However, the application of PRO measures faces numerous challenges. The statistical properties of patient-reported ordinal functional scales for rare diseases have been criticised due to their reliance on small, heterogeneous cohorts for development, and thus may be less sensitive to functional changes [36]. Furthermore, person or caregiver reporting measures face inherent biases as responses may be biased by the respondent’s personal, environmental and socio-cultural factors, such as culture, gender and location. This is especially relevant for prompts regarding an individual’s everyday experience, such as self-care, mobilising and communication. As such, PRO measures must be constructed and interpreted with an understanding of the diversity of the SMA community, and how participants may compare to a validation group [64]. The use of multi-informed assessment in clinical environments, for example, synthesising self and caregiver-reported PRO measures with performance-based assessments and clinical evaluation, is essential in harnessing the strengths of PRO and performance-based measures while mitigating their limitations [37]. Discrepancies in perspectives between self-reported and caregiver-reported outcomes are established and can enrich insights into family burden and unnoticed distress to provide a more complete assessment of health and context [31].

**Fig. 3:**
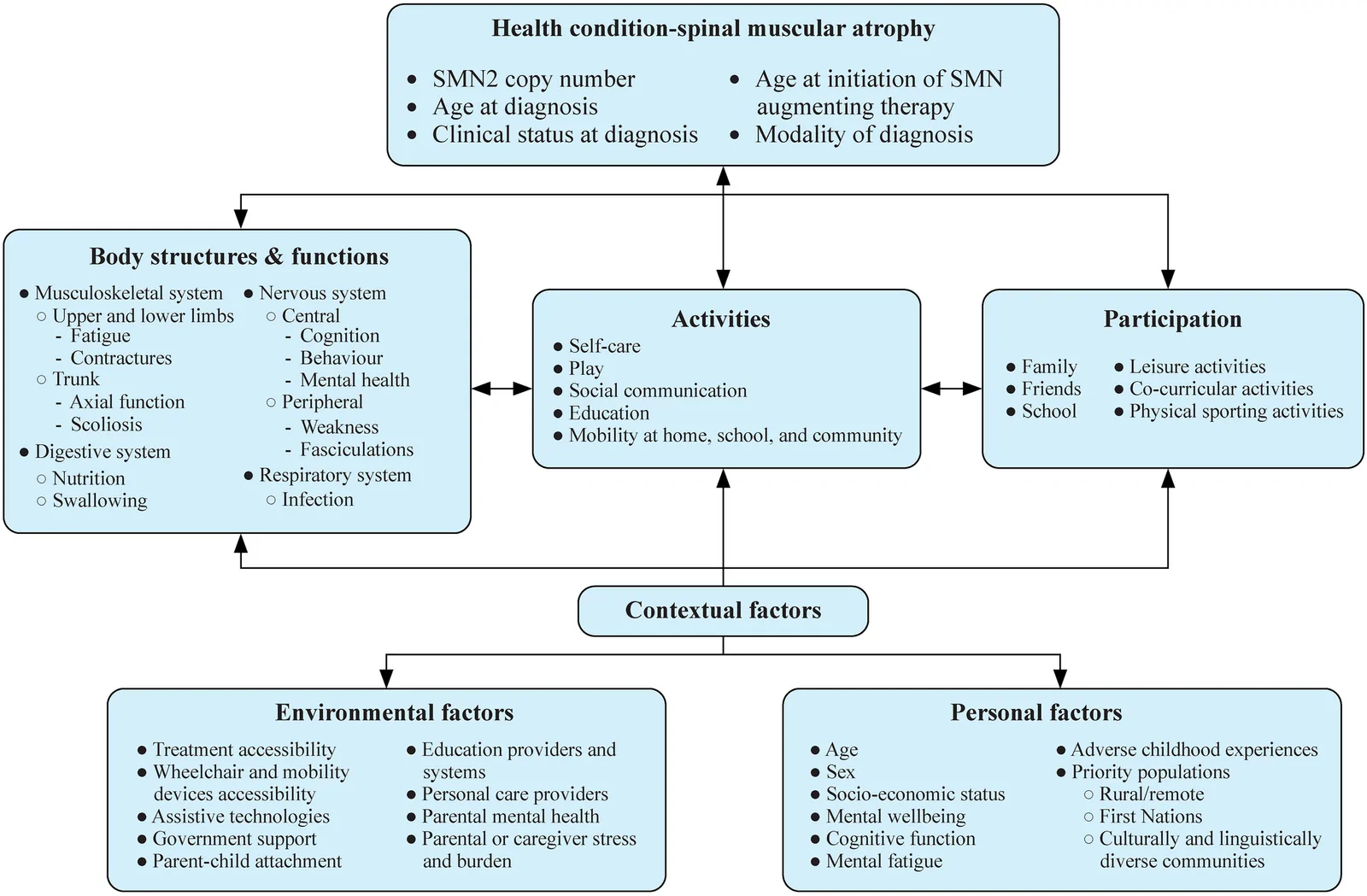
Application of the International Classification of Functioning, Disability and Health (ICF) model to spinal muscular atrophy (SMA). Adapted from the World Health Organization (WHO). This is an adaptation of an original work “ICF: International classification of functioning, disability and health. Geneva: World Health Organization (WHO); 2001. Licence: CC BY-NC-SA 4.0”. This adaptation was not created by the WHO. WHO is not responsible for the content or accuracy of this adaptation. The original edition shall be the binding and authentic edition. *SMN* survival motor neuron

The development of SMA-specific PRO measures in alignment with the ICF can ensure meaningful outcomes are assessed and thus inform future goal-setting and clinical priorities. However, the lack of validated, disease-specific PRO measures which assess adaptive functioning in children with SMA poses challenges in capturing these outcomes [37]. Future SMA PRO tools which capture adaptive functioning must be appropriate and efficient, capturing the factors elicited from formative research (fatigue, pain, scoliosis, function, contractures, feeding, breathing weakness, socio-communicative difficulties), in alignment with the ICF framework [9, 31, 65] (Fig. 3). Validation of PROs involves Rasch analysis to determine the optimal number of items and scoring formats across the spectrum of phenotypes, and subsequent assessment of external validity, reliability and sensitivity to meaningful changes related to functional independence. Developing and integrating such measures clinically will foster assessment of adaptive functioning in cohorts of children with SMA who have received SMN augmenting therapies, enabling efficient stratification for children in need of early intervention [36].

### Limitations

The limitations of current evidence include small sample studies, with a cross-sectional and single centre design and emphasis on specific phenotypes, limiting the reliability and generalisability of results. Development of a systematic approach that includes all phenotypes, ages, modalities of diagnosis and treatment is required to provide a broader understanding of adaptive functioning. Further studies in different healthcare and cultural settings are also warranted.

### Recommendations for practice

**Fig. 4:**
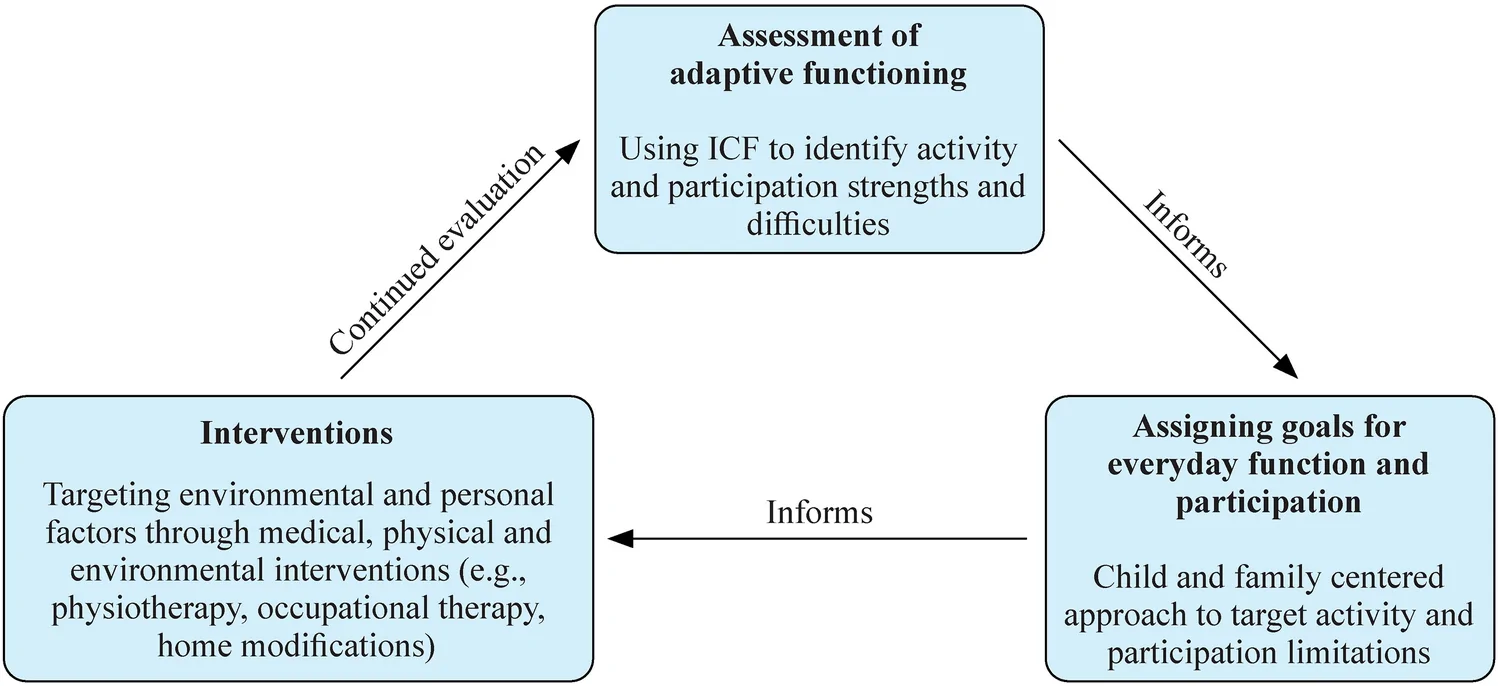
Operalisationizing the International Classification of Functioning, Disability and Health (ICF) to address adaptive functioning in spinal muscular atrophy (SMA). *ICF* The International Classification of Functioning, Disability and Health

Implementing the ICF as a biopsychosocial model is an important step in foregrounding adaptive functioning and participation for SMA care and providing a common language for caregivers, medical practitioners and multidisciplinary care providers to facilitate clear communication and child and family-centred goal setting. Operationalising the ICF within the neuromuscular clinic requires familiarity with the core terms of the ICF and an understanding of how this framework can be applied to children with SMA, as outlined in Fig. 3. Practical implementation of the ICF framework can be separated and applied into three core actions; assessment of activity and participation, child- and family-centred goal setting and prescription of interventions (targeting activity limitations and environmental influences) (Fig. 4). Assessment of activity and participation should incorporate both PRO and performance-based measures with clinical evaluation to identify domains of strength and challenge alongside environmental influences. Multidisciplinary goal setting and the subsequent prescribing of corresponding interventions can be mapped using the ICF framework to consider how each will influence the child’s activity and participation.

In conclusion, as SMA care enters a new decade of progress following the introduction of SMN augmenting therapies, it is timely to reflect upon progress made, and plan how the next decade of SMA care will continue to evolve. Guided by the priorities of people with SMA, the implementation of adaptive functioning as assessed through the domains of the ICF framework is pertinent to prognosticate outcomes, tailor interventions and provide best-practice care. Therefore, as a phenotypically diverse cohort of children treated with SMN augmenting therapies emerges, it is timely for clinicians to consider a holistic approach to SMA to equip families in preparing for the child’s future functional capabilities.

## Data Availability

Not applicable (not original research).
